# A wheat cytochrome P450 enhances both resistance to deoxynivalenol and grain yield

**DOI:** 10.1371/journal.pone.0204992

**Published:** 2018-10-12

**Authors:** Lokanadha R. Gunupuru, Chanemougasoundharam Arunachalam, Keshav B. Malla, Amal Kahla, Alexandre Perochon, Jianguang Jia, Ganesh Thapa, Fiona M. Doohan

**Affiliations:** School of Biology & Environment Science and Earth Institute, University College Dublin, Science Centre East, Belfield, Dublin 4, Ireland; Universita degli Studi di Pisa, ITALY

## Abstract

The mycotoxin deoxynivalenol (DON) serves as a plant disease virulence factor for the fungi *Fusarium graminearum* and *F*. *culmorum* during the development of Fusarium head blight (FHB) disease on wheat. A wheat cytochrome P450 gene from the subfamily CYP72A, *TaCYP72A*, was cloned from wheat cultivar CM82036. *TaCYP72A* was located on chromosome 3A with homeologs present on 3B and 3D of the wheat genome. Using gene expression studies, we showed that *TaCYP72A* variants were activated in wheat spikelets as an early response to *F*. *graminearum*, and this activation was in response to the mycotoxic *Fusarium* virulence factor deoxynivalenol (DON). Virus induced gene silencing (VIGS) studies in wheat heads revealed that this gene family contributes to DON resistance. VIGS resulted in more DON-induced discoloration of spikelets, as compared to mock VIGS treatment. In addition to positively affecting DON resistance, *TaCYP72A* also had a positive effect on grain number. VIGS of *TaCYP72A* genes reduced grain number by more than 59%. Thus, we provide evidence that *TaCYP72A* contributes to host resistance to DON and conclude that this gene family warrants further assessment as positive contributors to both biotic stress resistance and grain development in wheat.

## Introduction

Cytochrome P450s are heme-containing membrane-bound enzymes that can perform several types of oxidation-reduction reactions [1]. They are involved in plant defence, secondary metabolite biosynthesis in the classical xenobiotic detoxification pathway [2,3]. There is compounding evidence to show that cytochrome P450s play a role in the host response to diseases, including the wheat response to Fusarium head blight (FHB) disease [4,5].

FHB is a devastating fungal disease of wheat, barley and other small grain cereals grown in warm and humid regions worldwide [6,7]. *Fusarium graminearum* (teleomorph: *Gibberella zeae*) is the principal causal agent of the disease. It infects wheat heads during flowering and thereby interferes with seed development leading to severe yield loss and reduced grain quality [7,8]. *F*. *graminearum* also produces trichothecene mycotoxins in infected grains, predominantly deoxynivalenol (DON), which are harmful to plant, human and animal health [9–11]. Some cereal varieties are resistant to FHB disease. DON was shown to induce cytochrome P450 genes in FHB-resistant genotypes of wheat and barley. Comparing wild type and *Tri5*^*-*^ *F*. *graminearum* DON-minus mutants, Boddu *et al*. [12] identified several cytochrome P450 transcripts that were specifically induced during trichothecene accumulation in barley plants. A cytochrome P450 gene, *CYP709C1*, associated with resistance to FHB and Fusarium seedling blight in wheat was highly induced in wheat spikelets inoculated with DON [13]. In barley, cytochrome P450s were induced in spikelets in response to DON application [14]. Walter *et al*. [4] showed that the transcript levels for two cytochrome P450s were significantly higher in progeny of a wheat population that inherited a genetic locus associated with FHB resistance as compared to those that did not. It remains to be determined whether cytochrome P450s are associated with detoxification of DON or the transformation of other disease/toxin-induced moieties. Indeed, there is no evidence that plant cytochrome P450s directly affect DON resistance, either via a role in detoxification or by other means. But recently, a bacterial cytochrome P450 was shown to catabolise DON to a derivative that was much less toxic to wheat [15].

Here we characterized the mycotoxin-responsive cytochrome P450 first identified by Walter *et al*. [4] and investigated its potential to respond to and improve DON and FHB resistance. The gene clustered within the CYP72A subfamily and is hereafter referred to as *TaCYP72A*. Gene expression studies investigated the effect of *F*. *graminearum* and the mycotoxin DON on the regulation of *TaCYP72A* homeologs in wheat. The effect of VIGS of *TaCYP72A* on the DON sensitivity of wheat was investigated. The VIGS experiment also assessed the contribution of *TaCYP72A* genes to grain development. Based on the results of this study, we describe the first wheat cytochrome P450 variants to positively contribute to both DON resistance and grain development.

## Materials and methods

### Plant and fungal material and propagation

*Triticum aestivum* (wheat) cultivars (cvs.) CM82036 (a cross between ‘Sumai-3’ and ‘Thornbird-s’) and Remus were used in this study. Wheat cv. CM82036 is resistant to FHB and DON, a trait associated with quantitative trait loci (QTL) located on chromosomes 3B and 5A [16]. Wheat cv. Remus is susceptible to FHB disease [16]. For wheat cultivation, seeds were germinated in darkness for 72 h at 24°C in 90 mm petri dishes containing moist Whatman No. 1 filter paper (Whatman, UK). The germinated seedlings were transferred to 3 litre pots containing John Innes compost No. 2 (Westland Horticulture, Dungannon, UK). Wheat studies were carried under contained glasshouse conditions with a day/night temperature regime of 25/18°C and light regime 16/8 h.

Wild type fungus *F*. *graminearum* strain GZ3639 and its DON-minus mutant derivative GZT40 were used in this study. Wild type GZ3639 is a DON-producing strain virulent on wheat heads, while the DON-minus mutant is less pathogenic [17]. The mutant is a derivative of GZ3639 in which *Tri5* gene was disrupted, thus preventing DON production [17]. Conidial inoculum (macroconidia) was produced in Mung bean broth [18] and was harvested, washed and adjusted to 10^6^ conidia/ml, all as previously described [19].

### Nucleic acid purification

DNA was extracted from flash-frozen plant tissue with the HP plant DNA mini kit (OMEGA) following manufacturers’ instructions. RNA from freeze-dried wheat heads was extracted as described previously [20], while RNA from leaf samples were extracted using the RNeasy plant kit (Qiagen, USA) according to the manufacturer’s instructions. DNase treatment of extracted total RNA was performed using the TURBO DNA-*free*TM kit (Ambion Inc., USA). The quality, yield and integrity of DNA and RNA were assessed as described previously [4].

### Gene cloning

The cDNA sequenced of *TaCYP72A-3A* from cv. CM82036 was obtained via 5’ rapid amplification of cDNA ends (RACE) using the GeneRacerTM kit (Invitrogen, UK) and gene-specific RACE primers (S1 Table). The gDNA sequence was determined for wheat cvs. CM82036 and Remus via PCR using gene-specific primers (S1 Table). PCR reactions were performed using 20 ng of DNA template, 0.25 μM each of forward and reverse primers in a 20 μl reaction containing 0.5 U Taq DNA polymerase and 1x PCR buffer (Invitrogen, UK), 1.5 mM MgCl_2_, and 125 μM of each dNTP. PCR reactions were conducted in a Peltier thermal cycler DNA engine (MJ Research, USA) and the PCR program constituted 94°C for 5 min, 30 cycles of 94°C for 5 s, 58°C for 45 s and extension of 72°C for 2 min, with a final extension at 72°C for 5 min.

### Phylogenetic analysis

BLASTn analysis of the wheat cv. Chinese spring genome (http://plants.ensembl.org and http://wheat-urgi.versailles.inra.fr) were used to determine the chromosomal location and identify wheat variants of *TaCYP72A-3A* (the cut-off was 90% identity and E value E<10^−50^). The open reading frame, intron splicing and acceptor sites of *TaCYP72A-3A* were deduced using NCBI ORF finder [21] and NetGene2 [22]. The CYP450 conserved domains were identified manually [23]. The deduced TaCYP72A-3A protein from cv. CM82036 was used to identify homologous sequences within other *Poaceae* via BLASTp analysis using Ensembl Plants (http://plants.ensembl.org; E value> 1e^-5^). The best hit obtained per species, along with TaCYP72A-3A from cvs. CM82036, Remus, Chinese Spring and other cv. Chinese Spring homeologs, were used to construct a Neighbor-Joining tree [24] using Molecular Evolutionary Genetics Analysis Version 7 software (MEGA7) (http://www.megasoftware.net) [25]. The bootstrap consensus tree was inferred from 10,000 replicates and the tree was drawn to scale, with branch lengths in the same units as those of the evolutionary distances used to infer the phylogenetic tree. The evolutionary distances were computed using the Poisson correction method within MEGA7 and are in the units of the number of amino acid substitutions per site. Sequence homology to other organisms was determined using BLASTn analysis within NCBI (blast.ncbl.nlm.nih.gov).

### Adult plant DON and FHB time course experiment

Adult plant DON and FHB time course experiments using wheat cv. CM82036 were as previously described by Perochon et al. [26]. At anthesis, two central spilelets per head were treated with either 0.02% Tween-20 (mock) or 16.87 mM DON in 0.02% Tween-20, while in a separate FHB experiment, the central spikelets were treated with either 20 μl 0.02% Tween-20 (mock) or this solution augmented with 2 x 10^4^ conidia of either *F*. *graminearum* strain GZ3639 (WT) or its non-DON-producing mutant derivative GZT40. Treated spikelets were harvested at various time points post-treatment. After harvest, the spikelets were flash-frozen in liquid N_2_ and stored at -70°C prior to RNA extraction. Both the DON and the FHB experiment each comprised two replica trials. In each trial, RNA was extracted form one pooled sample per treatment per time point (representing a pool of 4 heads from individual plants).

### Virus-induced gene silencing (VIGS) experiment

The barley stripe mosaic virus (BSMV)-derived VIGS vectors used in this study consisted of the wild type BSMV ND18 α, β and γ tripartite genome [27,28]. Silencing of *TaCYP72A* was performed using two independent overlapping gene fragments (S2 Fig). Fragments were amplified from the CDS of *TaCYP72A-3A* from wheat cv. CM82036 via PCR (see S1 Table for primer details). These fragments were selected and designed to target the 3A, 3B1, 3B2 and 3D genome homeologs of *TaCYP72A* (S2 Table). PCR reactions were performed with 20 ng plasmid DNA, 1 μM each of forward and reverse gene-specific primers in a 10 μl reaction containing 0.5 U Taq DNA polymerase and 1x PCR buffer (Invitrogen, UK), 1.5 mM MgCl_2_, and 125 μM of each dNTP. PCR reactions were conducted in a Peltier thermal cycler DNA engine (MJ Research, USA and the PCR program consisted of an initial denaturation step at 94 ^o^C for 2 min, 35 cycles of 94 ^o^C for 30 s and 60 ^o^C for 30 s and a final extension step at 72 ^o^C for 5 min. Amplicons were cloned into *Not*I-digested γ RNA vector, pSL038-1 [28]. A BSMV γ RNA construct containing a 185 bp fragment of the barley phytoene desaturase (PDS) gene served as a positive control for VIGS and has been previously described [28]. The BSMV RNA was prepared and inoculated onto flag leaves of wheat cv. CM82036 as described previously [26]. Treatments were mock buffer treatment (FES), RNA prepared from virus control (BSMV:00) or RNA targeting the silencing of *PDS* (BSMV:PDS4as) or *TaCYP72A* (BSMV:CYP1 and BSMV:CYP2). At mid anthesis (growth stage 65; [29], the florets of the central head spikelets from the BSMV-infected tillers were treated with 10 μl of either DON (5 mg ml^-1^ 0.2% Tween 20) or 0.2% Tween 20 (control) as described previously [26]. Treated heads were covered with plastic bags for 2 days to maintain high humidity. After 24 h, a spikelet directly above the treated spikelet was harvested, flash frozen in liquid N_2_ and stored at -70 ^o^C for gene expression studies. The number of bleached spikelets (including treated spikelets) was assessed at 14 days after DON treatment. At harvest (GS90) the number of grain per head were determined. Grain were dried and the average weight of a single grain was determined (per head). The VIGS experiment comprised two trials, each of which included 19 heads (9 plants) per treatment combination, arranged in a randomised layout (per trial, 16 were used for phenotyping /gene expression and 19 for yield analysis).

### Gene expression studies

Total RNA was extracted from plant material and DNase-treated as described by Ansari et al. [20]. First strand cDNA synthesis and real time RT-PCR analysis to quantify the accumulation of the chromosome 3A, 3B and 3D variants of the *TaCYP72A-840* gene were done as described in Walter *et al*. [4] using homeolog-specific primers (S1 Table; note the 3B specific-primers target both the homologs of on this chromosome). Gene expression was normalised to that of the constitutively expressed housekeeping genes (HK) α-tubulin (GenBank No. U76558.1) [30] and GAPDH (GenBank No. EF592180.1) [31] (see S1 Table for primer sequences). Real time quantification of the accumulation of *TaCYP72A* transcripts and of the housekeeping genes was performed in separate reactions. The threshold cycle (C_t_) values obtained by real-time RT-PCR were used to calculate the fold change in transcript accumulation with the formula 2 ^- (Ct target transcript–Ct Average HK)^ [32]. All real time RT-PCR results were based on at least two independent reactions per sample. *In silico* analysis was also conducted in order to extract gene expression data for *TACYP72A* homeologs from an FHB RNAseq experiment [33] (corresponding gene IDs: *TaCYP72A-3A = TraesCS3A01G532600; TaCYP72A-3B1 = TraesCS3B01G609400; TaCYP72A-3B2 = TraesCS3B01G609600*; *TaCYP72A-3D = TraesCS3D01G537800*).

### Statistical analyses

All the data analyses were conducted using MINITAB 16 (Minitab Ltd., Coventry, UK). Normal distribution of data sets was determined using the Ryan Joiner test [34] within Minitab. Non-normally distributed data sets were transformed to fit a normal distribution using the Johnson transformation [34] within Minitab and the statistical significance of variance incorporating Tukey’s test (*P* = 0.05). The data which could not be transformed using the Johnson transformation [34] was analysed using the non-parametric Mann-Whitney test. The homogeneity of variance between replicate data sets was confirmed by correlation analysis (*r* ≥ 0.901, *P ≤* 0.05, based on Spearman Rank for non-normal and Pearson for normally distributed data) and thus experimental data sets were analysed as one.

## Results

### Cloning *TaCYP72A* and phylogenetic characterisation of gene variants

**Fig 1:**
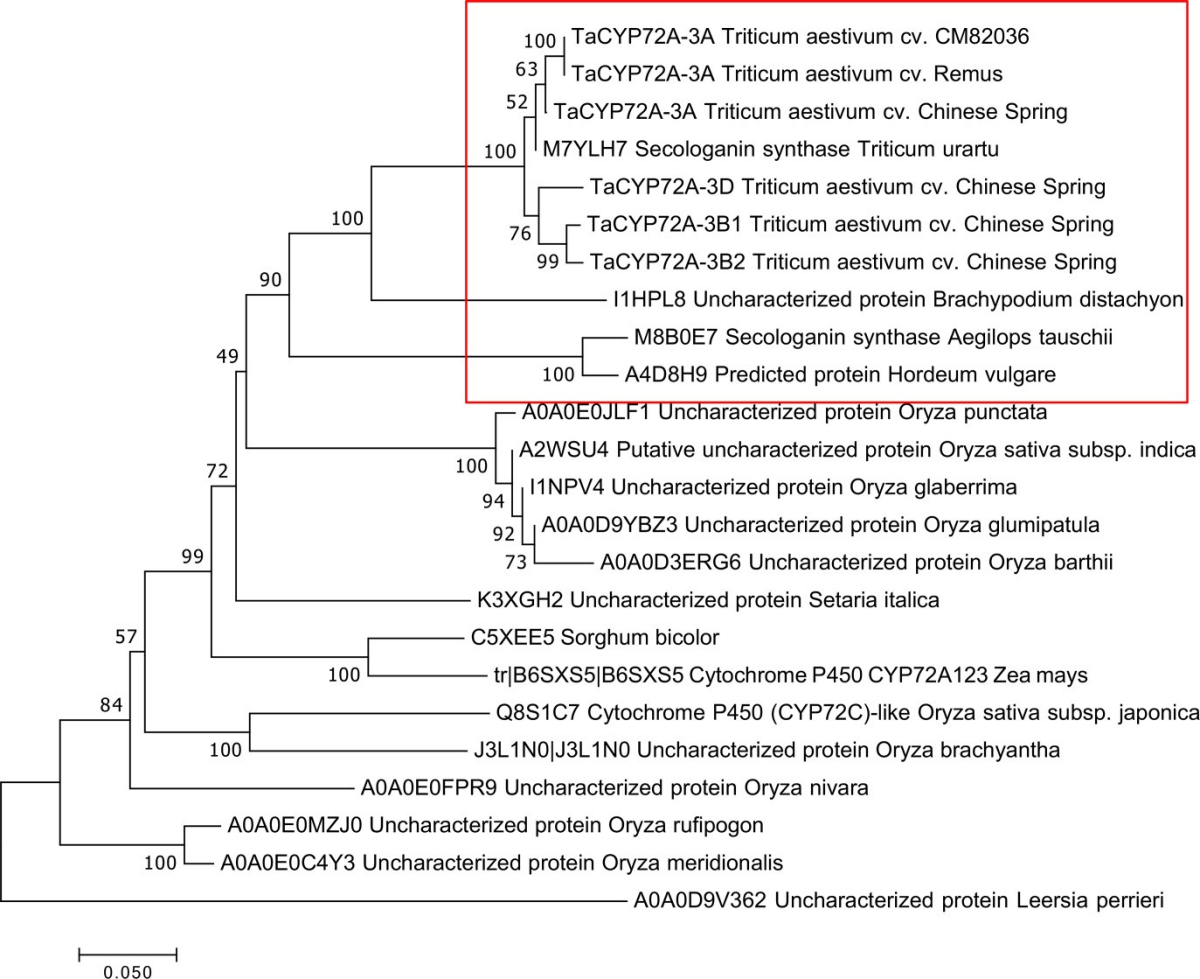
Phylogenetic analysis of *Ta*CYP72A and *Poaceae* homologs. The deduced amino acid sequences of wheat cv. Chinese Spring *TaCYP72A* genes, the 3A homologs from cvs. CM82036 and Remus, and the closest *Poaceae* CYP72A sequences obtained from Ensembl Plants and URGI were used for phylogenetic analysis. Evolutionary distances were computed using the Poisson correction method and are in the units of the number of amino acid substitutions per site. The Neighbor-Joining tree was constructed using Molecular Evolutionary Genetics Analysis Version 7 software (MEGA7). The bootstrap consensus tree was inferred from 10000 replicates. The tree was drawn to scale, with branch lengths in the same units as those of the evolutionary distances used to infer the phylogenetic tree. The red box denotes wheat and other *Pooideae* proteins.

Previous studies within our laboratory identified a novel cytochrome wheat transcript that was responsive to the *Fusarium* mycotoxin DON (referred to as *TaCYP72A-*840) [4,5]. We sequenced and compared the mRNA and gDNA sequences from bread wheat cv. CM82036 and thus deduced that the gene contains three introns and four exons (S1A Fig). The gene was named *TaCYP72-3A* because when compared to the sequenced wheat cv. Chinese Spring genome, the gene is almost identical to a gene (TraesCS3A01G532600) on chromosome 3A (99.34%; S3 and S4 Tables). The two main differences are that the cv. Chinese Spring gene has a different start codon position and one intron is larger (an additional 3.5 kb) relative to cv. CM82036, (S1A Fig and S4 Table). We also sequenced the *TaCYP72A-3A* gene from wheat cv. Remus and it was similar to that of cv. CM82036 (99% identity) (S1A Fig and S4 Table). The sequenced genome of cv. Chinese Spring encodes three other homeologs of the gene. There are two variants on the 3B genome (3B1 and 3B2) and one on chromosome 3D (S1B Fig; S3 and S4 Tables). The encoded proteins respectively share 95.2, 94.7 and 94.7% homology with *TaCYP72A* from cv. CM82036. The *Poaceae* proteins homologous to *TaCYP72A* are annotated in NCBI as either CYP72A proteins or proteins of unknown function with CYP domains (Fig 1). The cvs. CM82036 and Remus *TaCYP72A* genes and the cv. Chinese Spring 3B1, 3B2 and 3D homeologs encode the typical cytochrome P450 conserved domains (transmembrane anchor, proline-rich region (often PPGP), C-helix, oxygen binding I-helix, K-helix, heme binding domains) (S1B Fig). The exception is that the cv. Chinese Spring 3A protein lacks the transmembrane anchor (S1 Fig).

Phylogenetic analysis deduced that the *Ta-CYP72A* wheat variants formed a distinct clade with two subgroups (Fig 1). Subgroup 1 comprised the 3A variants from cvs. CM82036, Remus and Chinese Spring and the gene from the wheat genome A progenitor, *Triticum urartu*, which has been annotated as a CYP72A1 encoding a secologanin synthase. Subgroup 2 comprised the 3B1, 3B2 and 3D homeologs from cv. Chinese Spring. Outside wheat, the closest *Poaceae* homologs were those from *Brachypodium distachyon*, *Aegilops tauschii* and *Hordeum vulgare* (Fig 1).

### *TaCYP72A* genes are mycotoxin-responsive

**Fig 2:**
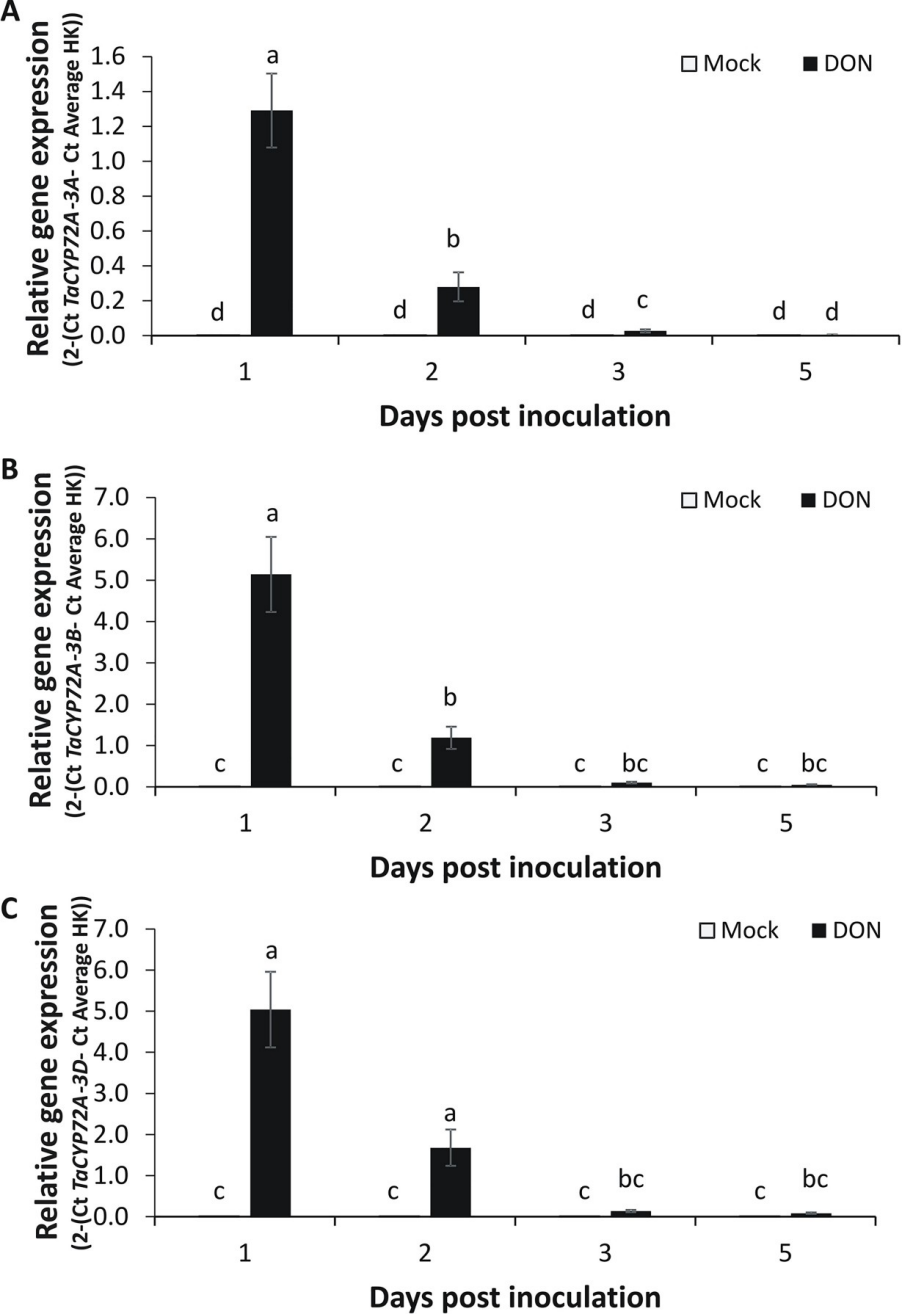
The accumulation of *TaCYP72A* transcripts in heads of wheat cultivar CM82036 in response to DON. Homeologs: (A) *TaCYP72A-3A* (B) *TaCYP72A-3B1/3B2* (the RT-PCR targeted both 3B genes) (C) *TaCYP72A-3D*. At mid anthesis (growth stage 65) [29] two central spikelets of the heads were inoculated with 20 μl (40 μl per head) of either DON (Santa Cruz, Texas, USA) (16.87 mM DON in 0.02% Tween 20,) or mock (0.02% Tween 20). Treated spikelets were harvested at 0, 1, 2, 3, 5 days after inoculation. RNA was extracted from the harvested spikelets and used for qRT-PCR analysis. Gene expression was quantified relative to wheat *α-tubulin* and *GAPDH* housekeeping (HK) genes (2 ^- (Ct *TaCYP72A –*Ct Average HK)^). Results represent the mean data obtained from two biological replicates, RNA was extracted from one pooled sample per treatment (representing a pool of 4 heads from individual plants) and bars indicate SEM. Columns with the same letter are not significantly different (*P<*0.05).

**Fig 3:**
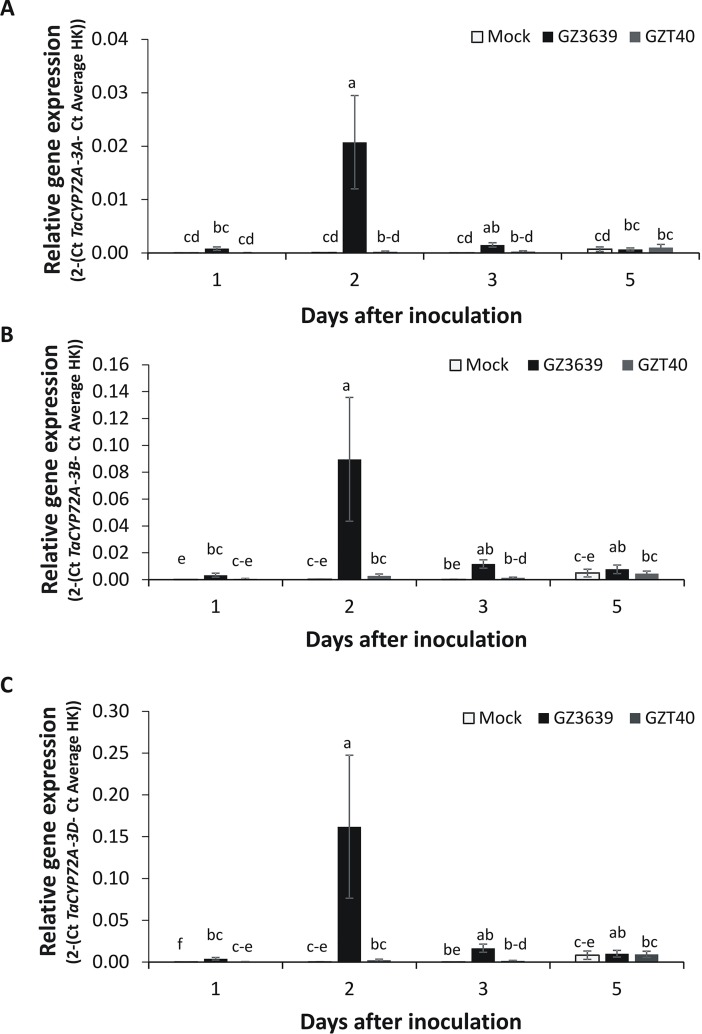
The accumulation of *TaCYP72A* transcripts in heads of wheat cultivar CM82036 in response to *F*. *graminearum*. Homeologs: (A) *TaCYP72A-3A* (B) *TaCYP72A-3B1/3B2* (the RT-PCR targeted both 3B genes) (C) *TaCYP72A-3D*. At mid anthesis (growth stage 65) [29] two central spikelets of the heads were inoculated with 2x10^4^ spores of either wild type *F*. *graminearum* GZ3639 or its DON-minus mutant derivative GZT40 or mock (0.02% Tween 20). Treated spikelets were harvested at 0, 1, 2, 3, 5 days after inoculation. RNA was extracted from the harvested spikelets and used for qRT-PCR analysis. Gene expression was quantified relative to wheat *α-tubulin* and *GAPDH* housekeeping (HK) genes (2 ^- (Ct *TaCYP72A –*Ct Average HK)^). Results represent the mean data obtained from two biological replicates, RNA was extracted from one pooled sample per treatment (representing a pool of 4 heads from individual plants) and bars indicate SEM. Columns with the same letter are not significantly different (*P<*0.05).

*TaCYP72A-3A* was originally identified as a DON-responsive gene in the FHB and DON-resistant wheat cv. CM82036 [4]. We used quantitative real-time RT-PCR (qRT-PCR) to analyse the temporal response of *TaCYP72A* variants to DON and *F*. *graminearum* in heads of ‘CM82036’, using primers specific to either (i) the chromosome 3A, (ii) both chromosome 3B variants (3B1 and 3B2) or (iii) to the 3D homeolog (Figs 2 and 3). In mock-treated tissue, the basal expression of the *3A*, *3B1/3B2* and 3D homeologs of *TaCYP72A* was near detectable limits, in contrast to the high level of gene expression in DON-treated tissues. DON induction of all *TaCYP72A* variants peaked at 1 day post-treatment (Fig 2). Thus, *TaCYP72A* genes were activated as part of the early response to the toxigenic *Fusarium* virulence factor DON. Based on their DON-responsiveness, we hypothesised that *TaCYP72A* homeologs would be activated as an early response to *Fusarium*. This was the case, with *F*. *graminearum* up-regulating transcription as early as 1 days post inoculation (dpi), induction peaking at 2 dpi and diminishing thereafter (Fig 3). *In silico* analysis of gene expression data from an FHB experiment showed that all *TaCYP72A* chromosomal variants were also responsive to *F*. *graminearum* in four other wheat genotypes at both 2 and 4 dpi (S3 Fig). At 2 dpi the chromosome 3A variant was more responsive to the pathogen in both the FHB resistant cv. Nyubai and its derivative HC374 than in the FHB resistant cv. Wuhan 1 or the FHB susceptible cv. Shaw. Notably, cv. Nyubai and its derivative HC374 carry the same cv. Sumai 3-derived FHB resistance QTL as does cv. CM82036 [34].

To determine if *Fusarium* activation of gene expression was toxin-dependent, we assessed the effect of a non-DON-producing mutant derivative of *F*. *graminearum* on *TaCYP72A* transcription. Unlike the wild type strain, the mutant had little effect on *TaCYP72A* expression (Fig 3).

Comparing the homeologs, we found that they all displayed a similar expression profile in response to DON (Fig 2), wild type *F*. *graminearum* and its’ DON-minus mutant derivative (Fig 3). The 3B and 3D homeolog expression levels were three times higher than that of the 3A homeolog in DON and *Fusarium-*treated cv. CM82036 samples at 1 dpi. But, the responsiveness of the 3B and 3D homeologs to DON and *Fusarium*, relative to mock treatment (i.e. the fold change), was similar to that of 3A (and the variants generally showed similar responses to *F*. *graminearum* in four other wheat cultivars based on the *in silico* analysis presented in S3 Fig [34]).

### Silencing of *TaCYP72A* reduces DON tolerance in wheat

**Fig 4:**
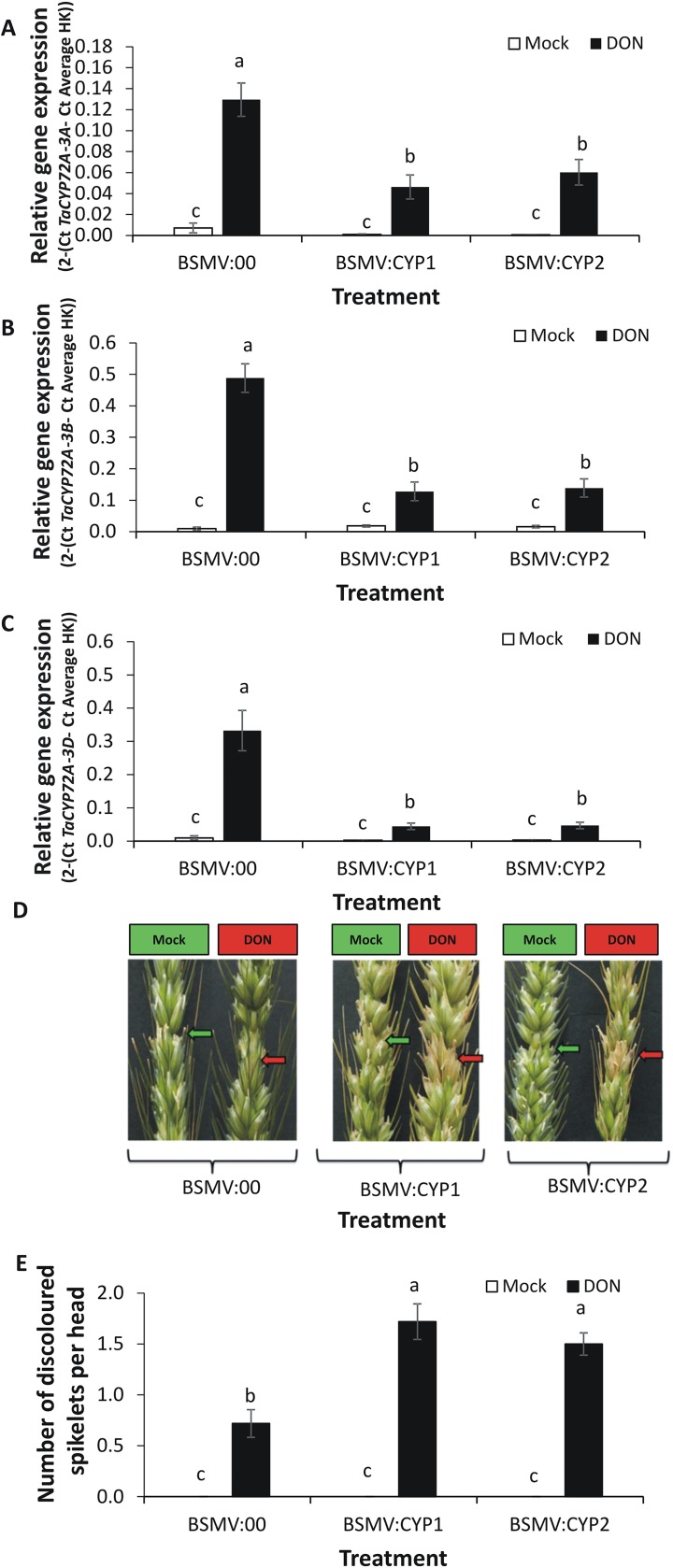
Virus-induced gene silencing (VIGS) of *TaCYP72A* in wheat heads. Plants of wheat cultivar CM82036 were subjected to VIGS using barley stripe mosaic virus (BSMV) constructs. Plants were treated with either BSMV:00 (empty vector) or BSMV:CYP1 or BSMV:CYP2 (constructs targeting *TaCYP72A*). Flag leaves were treated with the tripartite BSMV plasmids [28], prior to emergence of the first head, and subsequently emergent head treated with either 10 μl of 5 mg ml^-1^ DON or 0.02% Tween 20 (Mock treatment) at mid-anthesis. The spikelets just above the treated spikelets were collected for gene expression studies. Gene silencing in wheat spikelets was quantified by qRT-PCR analysis using primers specific to *TaCYP72A-840* homeologs on chromosome (A) 3A, (B) 3B1/3B2 and (C) 3D; expression was calculated relative to the reference genes *α-tubulin* and *GAPDH* (2 ^- (Ct *TaCYP72 –*Ct Average HK)^). (D) By 14 dpi DON-induced discolouration was more evident on gene-silenced as compared to on mock (virus) treated samples (arrow indicates spikelets treated with either Tween 20 or DON). (E) Quantification of the DON-induced discolouration of spikelets. Note that BSMV:00 treatment did not itself change the phenotype (as compared to non viral FES buffer treatment, for which the phenotypic results were previously shown [35]. Results (A), (B), (C) and (E) are the average of 32 heads per treatment (from two biological replicates). Error bars indicate SEM. Columns with the same letter are not significantly different (*P*<0.05).

DON is phytotoxic. Wheat genotypes vary in their ability to tolerate the toxin and, when it is applied to intolerant heads, it causes damage in the form of bleached spikelets [4]. A virus-induced gene silencing (VIGS) experiment was conducted to determine if *TaCYP72A* homeologs contribute to DON tolerance in wheat cv. CM82036. Silencing was achieved using two constructs (BSMV:CYP1 and BSMV:CYP2; S2 Fig), applied as independent treatments, and these targeted all four homeologs of the gene (3A, 3B1, 3B2 and 3D). Gene expression analyses (qRT-PCR) specific to each of the homeologs was conducted in order to validate the efficacy of VIGS. DON treatment of central head spikelets induced *TaCYP72A* expression, but in gene-silenced plants the DON induction of *TaCYP72A-3A* was significantly reduced by 62 and 54%, respectively, by treatment with BSMV:CYP1 and BSMV:CYP2, as compared to the effect of DON on plants treated with the mock virus (BSMV:00) (*P <* 0.05; Fig 4A). Gene silencing was also observed for the 3B1/3B2 and 3D homeologs. Treatment with BSMV:CYP1 and BSMV-CYP2 resulted in a 71–88% decrease in the accumulation of the chromosome 3B and 3D transcripts in DON treated heads, as compared to the DON effect in heads treated with the mock virus (BSMV:00) (*P <* 0.05; Fig 4B and 4C). In the absence of DON, minimal *TaCYP72A* expression was observed, but expression was usually lower (albeit not significantly so) in gene silenced as compared to non-silenced plants.

At a phenotypic level, assessment of heads at 14 days post-toxin treatment showed that BSMV:CYP1 and BSMV:CYP2 treated plants were significantly more sensitive to DON-induced damage than the BSMV:00 treated plants (*P <* 0.05; Fig 4D and 4E). Silencing of *TaCYP72A-840* variants resulted in > 2.3-fold increase in the number of DON-damaged spikelets (in BSMV:CYP1 or BSMV:CYP2 versus BSMV:00 plants). Cultivar CM82036 is very resistant to DON-induced bleaching of heads and plants treated with BSMV:00 showed very little discolouration, i.e. less than one spikelet per head showed a brown discolouration, the average being 0.6 (Fig 4D and 4E). Those treated with either BSMV:CYP1 or BSMV:CYP2 showed up to 1.5 spikelets per head discoloured due to the toxin, the average being 1.6 and 1.4, respectively.

### *TaCYP72A-3A* positively effects grain number and reduces yield loss due to DON

**Fig 5:**
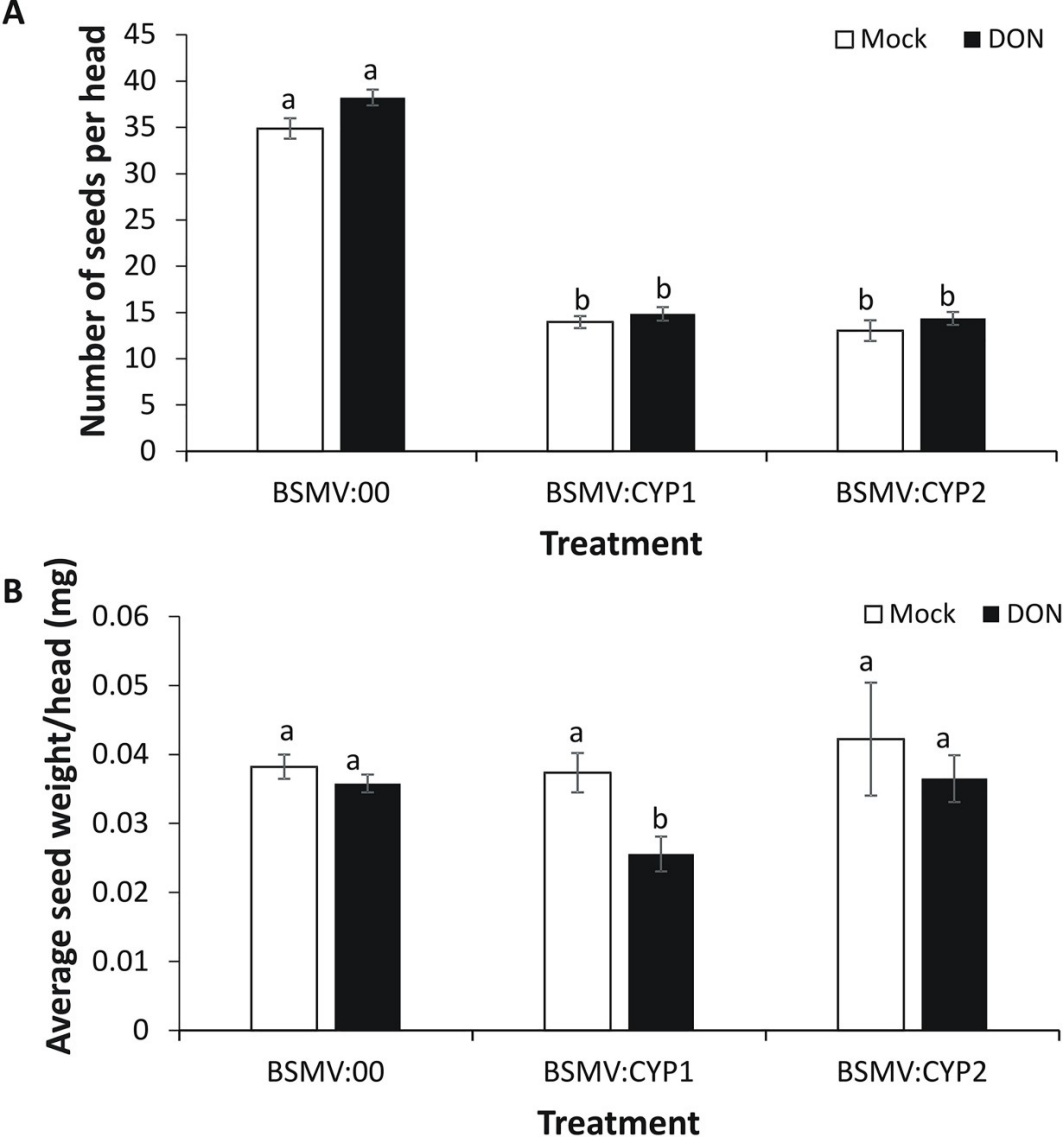
Effect of silencing *TaCYP72A* genes on grain number and weight in both mock and DON-treated heads of wheat cultivar CM82036. Virus induced gene silencing (VIGS) was conducted as described in Fig 4 (flag leaf VIGS treatments being empty virus BSMV:00 or treatments BSMV:CYP1 and BSMV:CYP2 which target *TaCYP72A* variants for silencing); subsequently, emergent heads were treated with 10 μl of 5 mg ml-1 DON or 0.02% Tween 20 (mock treatment). At harvest the heads were collected and both the (A) grain number and (B) average grain weight per head were determined. Results are the average of 32 heads per treatment (from two biological replicates). Error bars indicate SEM. Columns with the same letter are not significantly different (*P*<0.05).

Although, the reductions in *TaCYP72A* gene expression due to VIGS in mock Tween 20 treated heads were not significant in the spikelets tested (Fig 4), it did affect grain number. VIGS of this gene subfamily did reduce the seed number obtained per head by >59% (*P*<0.05) compared with plants treated with empty virus BSMV:00. This reduction was observed in both mock Tween 20 and toxin-treated heads and was therefore not dependent on DON (Fig 5A). Indeed, comparing DON and Tween 20 treatment in BSMV:00-treated tissue, it was evident that DON treatment did not significantly reduce either grain number (Fig 5A) or grain weight (Fig 5B). But, gene silencing with either BSMV:CYP1 and BSMV:CYP2 exacerbated the negative effect of DON on grain weight, and significantly so for the former construct (31% reduction in grain weight in BSMV:CYP1-treated plant when comparing DON versus Tween 20 treated heads; Fig 5B). Overall the results led us to conclude that *TaCYP72A* positively influences grain development.

## Discussion

This study confirmed that *TaCYP72A* from the CYP72A subfamily of cytochrome P450s contributes to DON resistance in wheat. Using the RNA from the same time course experiment as used herein, Perochon et al. [26] recently reported that the transcription of the *Fusarium* DON biosynthetic gene *FgTri5* peaked at 2 dpi, and this coincides with the peak in pathogen induction of *TaCYP72A*. Thus, we concluded that the expression of *TaCYP72A* was coincident with toxin biosynthesis. Perochon et al. [26] also reported that both the DON-minus mutant and the wild type *F*. *graminearum* induced a defence response in wheat, as determined via qRT-PCR analysis of the defence marker gene *Triticum aestivum* Pathogenesis-Related-1 (*TaPR1*) [26]. Hence, since *TaCYP72A* was only induced by the wild type fungus and not the mutant, we conclude that *TaCYP72A* is activated as part of the wheat response to toxin production rather than as part of a general defence response against *F*. *graminearum*.

Cytochrome P450s of CYP72 family belong to the non-A type cytochrome P450s which include highly divergent group of sequences that show local similarity to non-plant cytochrome P450s than to other plant cytochrome P450s and function in lipid or hormone metabolism while the A-type P450s are involved in biosynthesis of secondary metabolites [36]. The CYP72A subfamily from the model plant *Arabidopsis* comprises a cluster of 8 cytochrome P450s, however their functions are still unknown [23]. The enzymatic function of *TaCYP72A* remains unknown, with no close homolog being biochemically characterised. Several homologs have been annotated as secologanin synthase proteins based on their homology to *Catharanthus roseus* protein CYP72A1. But the homology between this protein and TaCYP72A is 51% and we should not infer anything from this as it appears to be typical that even closely related CYP72A proteins are involved in different pathways, suggesting that CYP72A functional evolution is independent of the other CYP72As in each plant species [37]. Trichothecenes are potent inhibitors of eukaryotic protein synthesis and DON inhibits the peptidyl transferase activity in 60S ribosome subunit [38]. It may be that the induction of the *TaCYP72A* and its homeologs is a downstream response to the protein synthesis inhibitory activity of trichothecenes, as gene expression studies confirmed that *TaCYP72A* is responsive to the protein synthesis inhibitor cycloheximide (unpublished data).

*TaCYP72A* is the first plant cytochrome P450 gene subfamily shown to enhance DON resistance in plants. This was validated via gene silencing in the plant of origin, wheat. *TaCYP72A* acts either to alleviate DON-induced stress or is directly involved in DON detoxification as a component of the classical xenobiotic detoxification pathway [39]. The increase in the number of DON-bleached spikelets in *TaCYP72A-*silenced wheat plants suggests that this gene subfamily might directly affect either DON detoxification or translocation. A wheat UDP-glucosyltransferase (*TaUGT3*) and a barley UDP-glucosyltransferase (*HvUGT13248*) have been cloned which were able to convert DON to DON-3-O-glucoside [40,41]. However, DON naturally possess a hydroxyl at the glucosylated C-3 atom [42,43] and thus there is no obvious need for cytochrome P450 activity to precede that of UGT. A cytochrome P450 enzyme from a DON-utilising bacterium, *Sphingomonas sp*., was shown to catabolise DON *in vitro* and the reaction product was identified as 16-hydroxy-DON [15]. In a bioassay using wheat seedlings, 16-hydroxy-DON showed reduced toxicity compared to DON [15]. This demonstrates that hydroxylation of DON is a DON detoxification mechanism and cytochrome P450s are capable of detoxifying DON independent of phase II enzymes. It is not known if a similar DON detoxification mechanism exists in plants. The bacterial enzyme and *TaCYP72A* belong to different subfamilies of cytochrome P450s.

The DON detoxification model proposed in animals by Sobrova *et al*. [39] suggests that cytochrome P450s might oxidise the free hydroxyl groups of DON to form DON radicals which might then be scavenged by antioxidant enzymes or conjugated with glutathione (GSH). The co-expression of wheat cytochrome P450 genes, including *TaCYP72A-840*, with UGTs, GSTs and ABC transporters [4,12,14] suggests that that plants might possess a variant of the proposed DON detoxification model in animals. The model suggests that cytochrome P450s might metabolise DON and the metabolites can be further conjugated with GSH for extrusion. DON-glutathione (GSH) conjugate was discovered as one of several DON conjugates in wheat [44]. That study also detected five other DON conjugates that remain to be characterised, thus highlighting the fact that we know very little about the DON biotransformation mechanisms that exist *in planta*. And recently, we found that a DON-responsive ABC family C drug transporter contributes to DON resistance in wheat. VIGS analysis of the encoding gene reduced wheat resistance to DON [35]. Thus there is compounding evidence for the activation and toxin-induced up-regulation of detoxification pathways in wheat. More importantly, there is evidence that they are important components of defence against DON.

Note that we also validated that overexpression of a variant of *TaCYP72A-3A* enhances FHB resistance in wheat (and positively affects grain development). This data is not presented because, unfortunately, based on the new wheat genome, we realised that there are three non-synonymous mutations in the overexpressed gene as compared to *TaCYP72A-3A* (but not in the active site). Nonetheless, collectively the results give us great confidence that *TaCYP72A* and variants thereof can enhance DON resistance.

While other subfamilies of CYP genes have been shown to influence both grain size and number [45,46], this is the first report that a CYP72A gene influences yield from wheat. This is the second gene that both enhances DON resistance and alters grain development; Walter et al. [35] showed that the DON-responsive ABC transporter *TaABCC3*.*1* enhanced DON resistance and affected grain formation. Indeed, based on a transcriptome study by Chetouhi et al. [47], they concluded that the molecular responses to FHB in a susceptible cultivar was congruent with those for grain development (at least in a susceptible genotype). Genes involved in the response to both FHB and grain development and the interaction of these two responses were enriched for those involved in GTP catabolic process, histone lysine methylation, response to growth hormone stimulus, embryo development, responses to pathogen and drug transmembrane transport. It is therefore without doubt that the ‘omics’ study of transgenics overexpressing gene such as *TACYP72A-840* will give insights into both FHB disease resistance and grain development. We are currently analyzing the allelic diversity of *TaCYP72A* across a broad array of wheat genetic stocks to determine if SNPs (Single Nucleotide Polymorphism) within the gene promoter or gene are associated with various traits. Working with breeders, we aim to determine if such markers are of benefit for FHB resistance breeding.

## Supporting information

S1 Fig **Alignment of the chromosome 3A variants of *TaCYP72A* from wheat cvs. CM82036 and Remus with chromosome 3A, 3B’s and 3D homeologs from wheat cv. Chinese Spring (abbreviated to CS)**. The DNA and protein sequences were aligned using multalin (http://multalin.toulouse.inra.fr/multalin/). (**A**) Schematic representation of the genomic DNA alignment from the start codon (ATG) to the stop codon (TAG). The introns (I-1, I-2 and I-3) and exons (E1, E2, E3 and E4). (**B**) Aligned protein sequences. The cytochrome P450 (CYP) conserved domains were identified manually [23]. The deduced amino acid sequences contained the cytochrome P450 conserved domains: transmembrane anchor, proline-rich region (often PPGP), C-helix (WVKHR), oxygen-binding I helix (A/G-G-X-E/D-T-T/S), K-helix (EVLR), P (E) R (F) clade signature and the heme-binding cysteine region (F-X-X-G-X-R-C-X-G).
(JPG)

S2 Fig **Illustration of the position of the VIGS fragments within the mRNA encoding the wheat *TaCYP72A* homeologs on chromosome 3A, 3B (two variants) and 3D targeted for gene silencing and the position of the qRT-PCR target used to validate VIGS efficacy**. Numbers indicate the nucleotide positions in the *TaCYP72A* mRNA and other homeologs sequences were based on the sequenced genome of cv. Chinese Spring. Illustrations are not to scale.VIGS1 = BSMV:CYP1, VIGS2 = BSMV:CYP2, qRT PCR = quantitative Real Time PCR, UTR = Untranslated region.
(JPG)

S3 Fig Fold change of wheat TaCYP72A homeologs in heads of four wheat cultivars in response to *Fusarium graminearum* (as compared to water-treated controls) at 2 and 4 days post-inoculation. Data for the TaCYP72A homeologs TraesCS3A01G532600 (*TaCYP72A-3A*), TraesCS3B01G609400 (*TaCYP72A-3B1*), TraesCS3B01G609600 (*TaCYP72A-3B2*) and TraesCS3D01G537800 (*TaCYP72A-3D*) *wa*s obtained from wheat RNA-seq experiments conducted by Pan et al. [34] and is presented as log_2_ fold change (*P*≤0.01 for all genotypes and time points, as compared to the water controls).
(PDF)

S1 Table Primers used in this study. (DOCX)

S2 Table Homology of VIGS constructs to wheat *TaCYP72A* homeologs from cv. Chinese Spring and sequenced 3A gene from cv. CM82036. (DOCX)

S3 Table *TaCYP72A* genes analysed in this study. (DOCX)

S4 Table DNA sequence similarity between the CDS of *TaCYP72A-3A* from wheat cv. CM82036 and homologs/homeologs from cvs. Remus and Chinese Spring. (DOCX)
