# Supplementary figures and images for: A wheat cytochrome P450 enhances both resistance to deoxynivalenol and grain yield
Source: PLoS One. 2018 Oct 12;13(10):e0204992. doi: 10.1371/journal.pone.0204992 (PMC6185721; doi:10.1371/journal.pone.0204992)

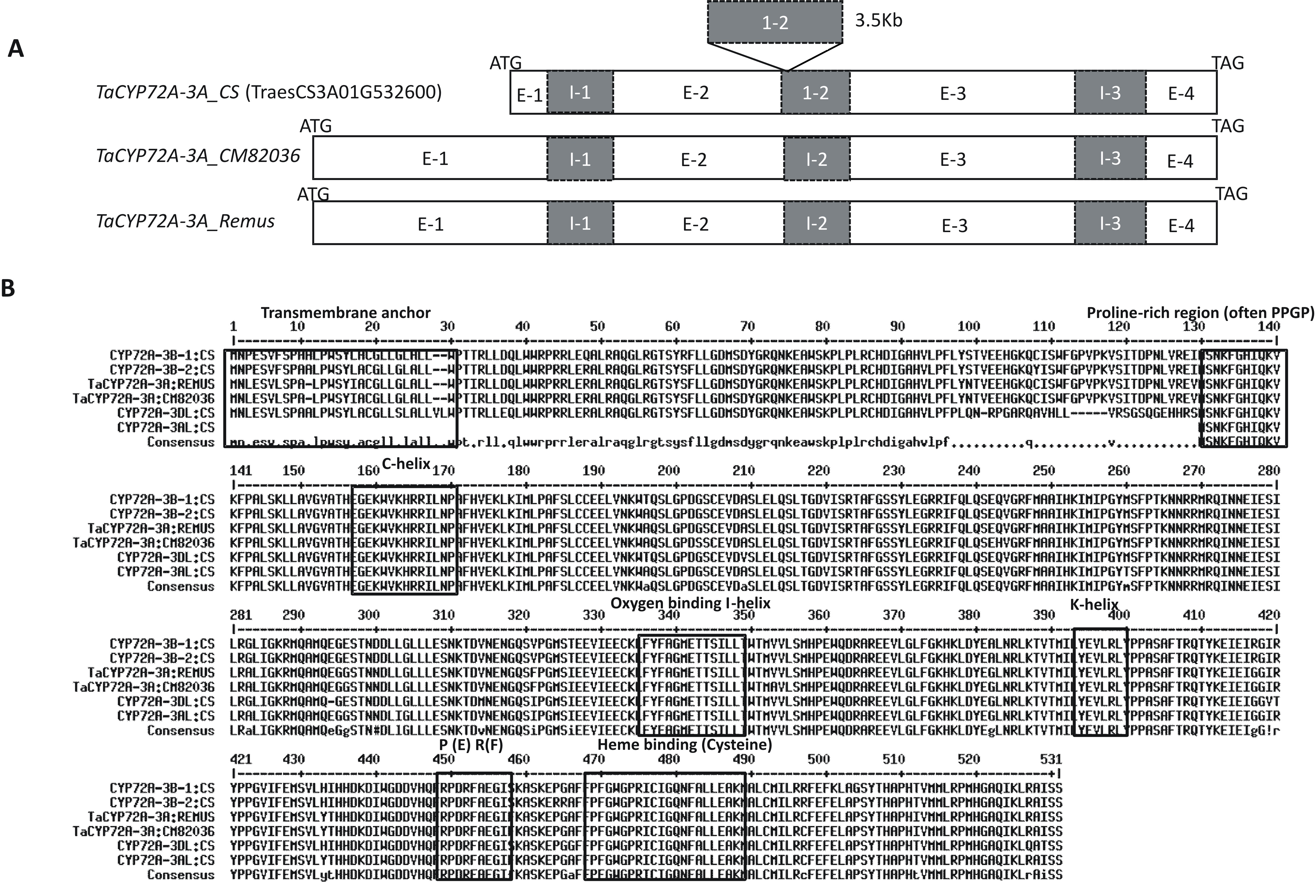

Supplement: S1 Fig — Alignment of the chromosome 3A variants of TaCYP72A from wheat cvs. CM82036 and Remus with chromosome 3A, 3B’s and 3D homeologs from wheat cv. Chinese Spring (abbreviated to CS). The DNA and protein sequences were aligned using multalin (http://multalin.toulouse.inra.fr/multalin/). (A) Schematic representation of the genomic DNA alignment from the start codon (ATG) to the stop codon (TAG). The introns (I-1, I-2 and I-3) and exons (E1, E2, E3 and E4). (B) Aligned protein sequences. The cytochrome P450 (CYP) conserved domains were identified manually [23]. The deduced amino acid sequences contained the cytochrome P450 conserved domains: transmembrane anchor, proline-rich region (often PPGP), C-helix (WVKHR), oxygen-binding I helix (A/G-G-X-E/D-T-T/S), K-helix (EVLR), P (E) R (F) clade signature and the heme-binding cysteine region (F-X-X-G-X-R-C-X-G). (JPG) [file pone.0204992.s001.jpg]

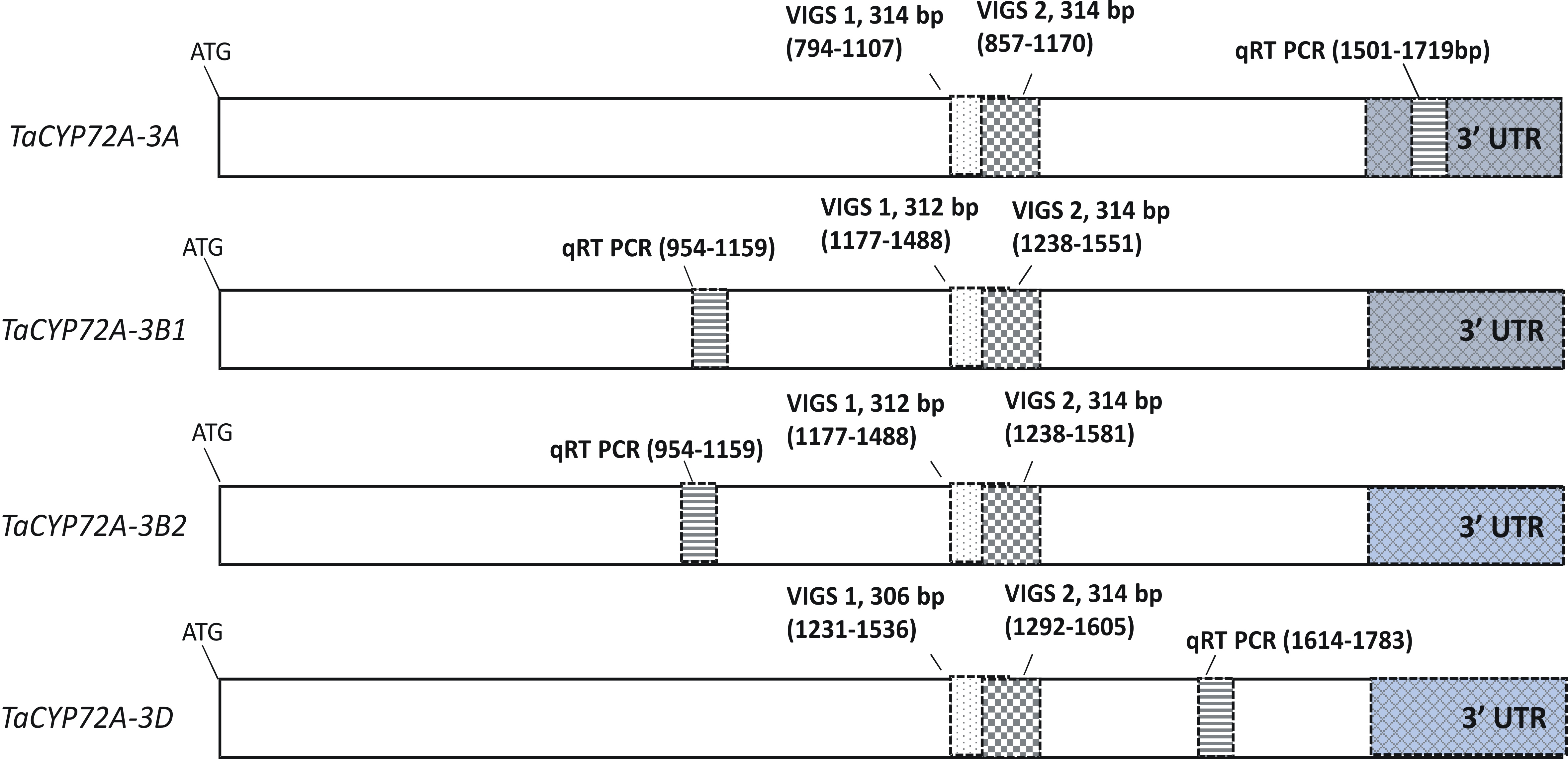

Supplement: S2 Fig — Illustration of the position of the VIGS fragments within the mRNA encoding the wheat TaCYP72A homeologs on chromosome 3A, 3B (two variants) and 3D targeted for gene silencing and the position of the qRT-PCR target used to validate VIGS efficacy. Numbers indicate the nucleotide positions in the TaCYP72A mRNA and other homeologs sequences were based on the sequenced genome of cv. Chinese Spring. Illustrations are not to scale.VIGS1 = BSMV:CYP1, VIGS2 = BSMV:CYP2, qRT PCR = quantitative Real Time PCR, UTR = Untranslated region. (JPG) [file pone.0204992.s002.jpg]

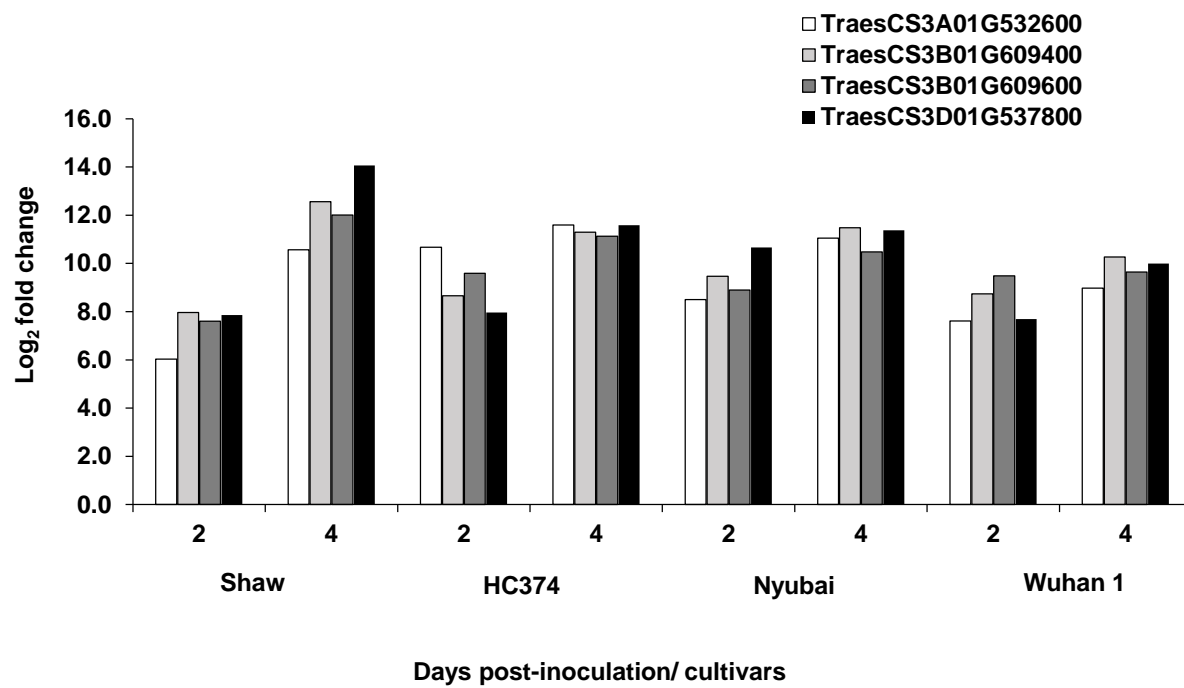

Fig. S3

Supplement: S3 Fig — Data for the TaCYP72A homeologs TraesCS3A01G532600 (TaCYP72A-3A), TraesCS3B01G609400 (TaCYP72A-3B1), TraesCS3B01G609600 (TaCYP72A-3B2) and TraesCS3D01G537800 (TaCYP72A-3D) was obtained from wheat RNA-seq experiments conducted by Pan et al. [34] and is presented as log2 fold change (P≤0.01 for all genotypes and time points, as compared to the water controls). (PDF) [file pone.0204992.s003.pdf]
