# Supplementary material for: A wheat cytochrome P450 enhances both resistance to deoxynivalenol and grain yield
Source: PLoS One. 2018 Oct 12;13(10):e0204992. doi: 10.1371/journal.pone.0204992 (PMC6185721; doi:10.1371/journal.pone.0204992)
Supplement: S1 Table — (DOCX) [file pone.0204992.s004.docx]

**S1 Table** Primers used in this study.

| **Primer name** | **Primer sequence (5' to 3')** | **Application** |
| --- | --- | --- |
| *TaCYP72A-3A* -RACE1 | ATGGGTGTACAACACGGACA | cDNA race 1 |
| *TaCYP72A*-*3A*-RACE2 | AACAAGCCGAGCAAGTCATT | cDNA race 2 |
| *TaCYP72A*-*3A*-RACE3 | GAAAGGATTGGCCATTTTCA | cDNA race 3 |
| *TaCYP72A-3A*-RACE4 | CTTGGACCATGCCTCTTTGT | cDNA race 4 |
| *TaCYP72A*-CDS:F/R | AATCTTGAATCCGTATTGAGTCC/ AGTATTACACCTTGTGAACGCC | Amplification of the CDS from gDNA of cvs. CM82036 and Remus |
| *TaCYP72A-*VIGS1:F/R | TTAATTAAGAGATCGGAGGCATCAGGTA/ CCCGGGCATGGGACGCAACATCATTA | Amplification of VIGS construct BSMV:CYP1 |
| *TaCYP72A-*VIGS2:F/R | TTAATTAACGAAGTTCTCCGGTTGTACC/ CCCGGGAAAGCACCGAAGAATCATGC | Amplification of VIGS construct BSMV:CYP2 |
| pGamma:F/R | TGATGATTCTTCTTCCGTTGC/  TGGTTTCCAATTCAGGCATCG | Sequencing BSMV:CYP1 and BSMV:CYP2 constructs |
| *TaCYP-3A*-RTPCR:F/R | CCGTCATATACTCACGCCC/  GCCCTGAATCATGTTGGAT | Real time RT-PCR of the *TaCYP72A* from chromosome 3A for time course and VIGS gene expression studies |
| *TaCYP-3B*-RTPCR:F/R | CGTTCCAGGAATGTCCACT/  CAGTTTTGAGTCGATTAAGAGCC | Real time RT-PCR of the two *TaCYP72A* from chromosome 3B for time course and VIGS gene expression studies |
| *TaCYP-3D-*RTPCR:F/R | TCAAATTAAGCTACAGGCGAC/ AATCATGTTGGACCTTTATTACG | Real time RT-PCR of the *TaCYP72* from chromosome 3D for time course and VIGS gene expression studies |
| *TaGAPDH*:F/R | TCACCACCGACTACATGACC/  ACAGCAACCTCCTTCTCACC | Real time RT-PCR housekeeping gene (Soltanloo et al., 2010) |
| *TaAlpha tubulin*:F/R | ATCTCCAACTCCACCAGTGTCG/  TCATCGCCCTCATCACCGTC | Real time RT-PCR housekeeping gene (Xiang et al., 2011) |
