# Supplementary material for: A wheat cytochrome P450 enhances both resistance to deoxynivalenol and grain yield
Source: PLoS One. 2018 Oct 12;13(10):e0204992. doi: 10.1371/journal.pone.0204992 (PMC6185721; doi:10.1371/journal.pone.0204992)
Supplement: S2 Table — (DOCX) [file pone.0204992.s005.docx]

**S2 Table** Homology of VIGS constructs to wheat *TaCYP72A* homeologs from cv. Chinese Spring and sequenced 3A gene from cv. CM82036.

| **VIGS construct** | **Percentage homology of VIGS constructs to *TaCYP72* homeologs** | | | | | |
| --- | --- | --- | --- | --- | --- | --- |
|  | **cv. CM82036** | | **cv. Chinese Spring** | | | |
|  | ***TaCYP72A-3A*** | | ***TaCYP72A-3A*** | ***TaCYP72A-3B1*** | ***TaCYP72A-3B2*** | ***TaCYP72A-3D*** |
| BSMV:CYP1 | 100 | | 99 | 94 | 94 | 91 |
| BSMV:CYP2 | 100 | 99 | | 96 | 95 | 92 |
