# Supplementary material for: A wheat cytochrome P450 enhances both resistance to deoxynivalenol and grain yield
Source: PLoS One. 2018 Oct 12;13(10):e0204992. doi: 10.1371/journal.pone.0204992 (PMC6185721; doi:10.1371/journal.pone.0204992)
Supplement: S3 Table — (DOCX) [file pone.0204992.s006.docx]

**S3 Table** *TaCYP72A* genes analysed in this study.

| **Wheat cultivar** | **Gene** | **Chromosome** | **Gene ID** | |
| --- | --- | --- | --- | --- |
|  |  |  | EnsemblPlants *^a^* | URGI*^b^* |
| CM82036 | *TaCYP72A-3A* | 3A |  |  |
| Remus | *TaCYP72A-3A* | 3A |  |  |
| Chinese Spring | *TaCYP72A-3A* | 3A | Not Annotated | TraesCS3A01G532600 |
| Chinese Spring | *TaCYP72A-3B1* | 3B | TRIAE_CS42_3B_TGACv1_224715_AA0800460 | TraesCS3B01G609400 |
| Chinese Spring | *TaCYP72A-3B2* | 3B | TRIAE_CS42_3B_TGACv1_221166_AA0732680 | TraesCS3B01G609600 |
| Chinese Spring | *TaCYP72A-3D* | 3D | TRIAE_CS42_3DL_TGACv1_249244_AA0842990 | TraesCS3D01G537800 |

*^a^*Gene ID extracted from website <http://plants.ensembl.org/Triticum_aestivum> ( EnsemblPlants *Triticum aestivum* TGAC v.1 browser).

*^b^*Gene ID extracted from website <https://urgi.versailles.inra.fr/> (IWGSC Reference Sequence v1.0 browser).
