# Supplementary material for: A wheat cytochrome P450 enhances both resistance to deoxynivalenol and grain yield
Source: PLoS One. 2018 Oct 12;13(10):e0204992. doi: 10.1371/journal.pone.0204992 (PMC6185721; doi:10.1371/journal.pone.0204992)
Supplement: S4 Table — (DOCX) [file pone.0204992.s007.docx]

**S4 Table** DNA sequence similarity between the CDS of *TaCYP72A-3A* from wheat cv. CM82036 and homologs/homeologs from cvs. Remus and Chinese Spring.

| **Wheat cultivar** |  |  | **Gene** | **Identity to *TaCYP-72A* from cv. CM82036 (%)** | **Query coverage**  **(bp)** |
| --- | --- | --- | --- | --- | --- |
| CM82036 |  |  | *TaCYP72A-3A* | 100 | 1-1587 |
| Remus |  |  | *TaCYP72A-3A* | 99.93 | 1-1587 |
| Chinese Spring |  |  | *TaCYP72A-3A* | 99.34 | 1-1209 |
| Chinese Spring |  |  | *TaCYP72A-3B1* | 95.15 | 1-1590 |
| Chinese Spring |  |  | *TaCYP72A-3B2* | 94.65 | 1-1590 |
| Chinese Spring |  |  | *TaCYP72A-3D* | 94.65 | 51-1647 |
